# Effect of Cromoglycate on Gas Changes, During Bronchial Challenge by UNCDW in Children with Asthma

**DOI:** 10.1155/S0962935194000748

**Published:** 1994

**Authors:** L. Mappa, S. Bavaro, G. Moramarco, D. Torio, D. Falciatore, G. Ciccarone, L. Cicciomessere, N. Rigillo

**Affiliations:** 1 Istituto di Pediatria Clinica e Sociale University of Bari Policlinico Piazza G. Cesare Bari 70124 Italy

## Abstract

Eighteen asthmatic children were challenged with ultrasonically nebulized cold distilled water (UNCDW). Blood gas composition was monitored transcutaneously (tcpO_2_ and tcpCO_2_) during and after the challenge. Assuming as basal the response to this UNCDW test, nine children (Group A) were then chosen at random to inhale cromoglycate by aerosol delivery for 8 days. Nine children (Group B), acting as a control, inhaled saline for 8 days. At the end of this therapy, each child repeated the UNCDW test. Statistical analysis with *t*-test for paired data was used to compare the results of each child to both tests. Mean basal tcpO_2_ and tcpCO_2_ were all within the expected normal range. In all children, both mean tcpO_2_ and tcpCO_2_ were reduced during and after UNCDW inhalation. Mean tcpCO_2_ values during the challenge were significantly (p < 0.001) lower than the corresponding steady state 2 rain after the UNCDW challenge, with a mean drop of −7% (2.1 S.D.). Mean tcpO_2_ values remained significantly decreased (p < 0.001) from the fifth mitt of the UNCDW challenge to the end of the observation period, with a mean drop of −20% (15.5 S.D.). After treatment with cromoglycate (Group A), the mean tcpCO_2_ values during UNCDW did not change significantly from those ofsteady state conditions: −0.8% (0.5 S.D.); whereas mean tcpO_2_ values decreased by −4% (4.9 S.D.). The control children treated with saline (Group B) showed mean tcpCO_2_ and tcpO_2_ values which were significantly different (p < 0.001) from those of the steady state conditions: mean drop of tcpCO_2_, −6% (4.2 S.D.); mean drop of tcpO_2_, −20% (4.7 S.D.). In conclusion, it emerges that: UNCDW induces nonspecific broncho-constriction in asthmatic children with a typical drop of tcpCO_2_ and tcpO_2_; the treatment with cromoglycate normalizes the time course of tcpCO_2_ (hyper-reactivity) and reduces dramatically the drop of tcpO_2_ time course (hyper-responsivity) during and after the UNCDW test.


Mediators of Inflammation 3, S39–S41 (1994)

MOIa14

### Effect of cromoglycate on gas changes, during bronchial challenge by UNCDW in children with asthma

L. Mappa,[CA] S. Bavaro, G. Moramarco, D. Torio, D. Falciatore, G. Ciccarone, L. Cicciomessere and N. Rigillo

Istituto di Pediatria Clinica e Sociale, University of Bari Policlinico, Piazza G. Cesare, 70124 Bari, Italy

[CA] Corresponding Author

**Eighteen asthmatic children were challenged with ultrasonically nebulized cold distilled water (UNCDW). Blood gas composition was monitored transcutaneously ($tcpO_2$ and $tcpCO_2$) during and after the challenge. Assuming as basal the response to this UNCDW test, nine children (Group A) were then chosen at random to inhale cromoglycate by aerosol delivery for 8 days. Nine children (Group B), acting as a control, inhaled saline for 8 days. At the end of this therapy, each child repeated the UNCDW test. Statistical analysis with *t*-test for paired data was used to compare the results of each child to both tests. Mean basal $tcpO_2$ and $tcpCO_2$ were all within the expected normal range. In all children, both mean $tcpO_2$ and $tcpCO_2$ were reduced during and after UNCDW inhalation. Mean $tcpCO_2$ values during the challenge were significantly ($p < 0.001$) lower than the corresponding steady state 2 min after the UNCDW challenge, with a mean drop of –7% (2.1 S.D.). Mean $tcpO_2$ values remained significantly decreased ($p < 0.001$) from the fifth min of the UNCDW challenge to the end of the observation period, with a mean drop of –20% (15.5 S.D.). After treatment with cromoglycate (Group A), the mean $tcpCO_2$ values during UNCDW did not change significantly from those of steady state conditions: –0.8% (0.5 S.D.); whereas mean $tcpO_2$ values decreased by –4% (4.9 S.D.). The control children treated with saline (Group B) showed mean $tcpCO_2$ and $tcpO_2$ values which were significantly different ($p < 0.001$) from those of the steady state conditions: mean drop of $tcpCO_2$, –6% (4.2 S.D.); mean drop of $tcpO_2$, –20% (4.7 S.D.). In conclusion, it emerges that: UNCDW induces nonspecific broncho-constriction in asthmatic children with a typical drop of $tcpCO_2$ and $tcpO_2$; the treatment with cromoglycate normalizes the time course of $tcpCO_2$ (hyper-reactivity) and reduces dramatically the drop of $tcpO_2$ time course (hyper-responsivity) during and after the UNCDW test.**

**Key words:** Asthma, Children, Cromoglycate, $O_2$ and $CO_2$ time course, Ultrasonic nebulized distilled water

### Introduction

Experience over the past 20 years has demonstrated the ability of cromoglycate to function as a mast cell stabilizer or inhibitor. Cromoglycate can be so considered among the anti-inflammatory drugs for asthma because it reduces bronchial hyper-reactivity[1] and decreases airway hyper-responsiveness.[2]

This study was designed to obtain further information on the effect of cromoglycate on the time courses of transcutaneous oxygen and carbon dioxide pressure ($tcpO_2$ and $tcpCO_2$), during bronchial challenge by ultrasonic nebulized 'cold' distilled water (UNCDW)[3,4] in children with asthma.

The possibility of performing continuous noninvasive monitoring of gas composition has been achieved by means of electrodes for transcutaneous $pO_2$ and $pCO_2$ monitoring.

This technique provides an accurate way of monitoring gas exchange changes during and after bronchial challenge.[5]

The bronchial challenge used is ultrasonically nebulized cold distilled water (UNCDW), which induces bronchoconstriction in children with asthma. The method is simple and reproducible, and is characterized by a high degree of specificity.[6]

### Materials and Methods

A placebo controlled study was undertaken on 18 asthmatic children (eight males, ten females) aged 3 to 12 years, with bronchial hyper-reactivity. At the time of the investigation, all children were free of symptoms and had not received any drug during the

© 1994 Rapid Communications of Oxford Ltd previous 8 days. Before submitting children to the study, informed consent was obtained from their parents.

Transcutaneous oxygen pressure ($tcpO_2$) and carbon dioxide ($tcpCO_2$) were monitored by the Gasthmatic system, after calibration, by placing the heated monoelectrode (44°C) on the upper chest of each child and allowing a stabilization period of 15–20 min. Baseline data were collected when the $tcpO_2$ and $tcpCO_2$ had reached a steady state level and the children were lying quietly without body movements.

The children were then given the UNCDW test: ultrasonic nebulizer De Vilbiss Ultra-neb 99 at 5 ml/min flow for 7 min was used. Cold distilled water was obtained by freezing it to around 0°C, and using it just at the time of testing. The $tcpO_2$ and $tcpCO_2$ values were recorded in the 5 min before the challenge, throughout the challenge procedures, and for 30 min following the end of nebulization.

For statistical purposes, comparisons were made between the mean values at the steady state level before nebulization and those measured during 0–5 and 15–30 min after nebulization. A $tcpO_2$ drop of over 15% below the baseline was taken as a positive response.

Assuming the response to UNCDW test as basal, nine children (Group A) were then chosen at random to inhale cromoglycate by aerosol delivery for 8 days (20 mg three times/day). Nine children (Group B) acted as placebo control, and inhaled saline (2 ml three times/day) for 8 days.

At the end of this therapy, each child repeated the UNCDW test. Statistical analysis with *t*-test for paired data was used to compare the results for each child for both tests.[7]

### Results

*Basal values:* The mean basal values for $tcpO_2$ and $tcpCO_2$ were all within the expected normal range. There were no significant differences in these baseline variables between the two groups of subjects.

*Response to UNCDW:* In all children, both mean $tcpO_2$ and $tcpCO_2$ were reduced during and after UNCDW inhalation. $TcpCO_2$ decreased immediately after the start of UNCDW exposure and the decrease was maximal around the third min of the challenge. The $tcpCO_2$ level started to pick up again immediately after the end of the exposure and the recovery was almost complete 5 min after the UNCDW test, with $tcpCO_2$ remaining more or less steady from that point onwards (Fig. 1).

The $tcpO_2$ time course was quite different, the decrease beginning at the fourth min of UNCDW inhalation and reaching the lowest value 4 min after the end of exposure (Fig. 2). Mean $tcpCO_2$ values during the challenge were significantly lower than the corresponding baseline ($p < 0.001$) 2 min after the UNCDW challenge, with a mean drop of −7% (2.1 S.D.). Mean $tcpO_2$ values remained significantly decreased ($p < 0.001$) from the fifth min of the UNCDW challenge to the end of the observation period, with a mean drop of −20% (15.5 S.D.).

After treatment with cromoglycate in children of Group A, the mean $tcpCO_2$ values during UNCDW were not significantly different from those of steady state (−0.8% (0.5 S.D.)), whereas mean $tcpO_2$ values decreased by −4% (4.9 S.D.).

The children treated with saline (Group B) showed mean $tcpCO_2$ and $tcpO_2$ values which were significantly different ($p < 0.001$) from those of the steady state (mean drop of $tcpCO_2$ −6% (4.2 S.D.)); mean drop of $tcpO_2$ −20% (4.7 S.D.)).

Comparisons made between $tcpCO_2/O_2$ data for each child of Group A to both tests (before and after therapy), showed a statistically significant difference ($p < 0.001$). Between data for each child of Group B, there were no statistically significant differences to both tests.

### Discussion

The possibility of monitoring blood gases with a non-invasive method (Gasthmatic) and the evalua-

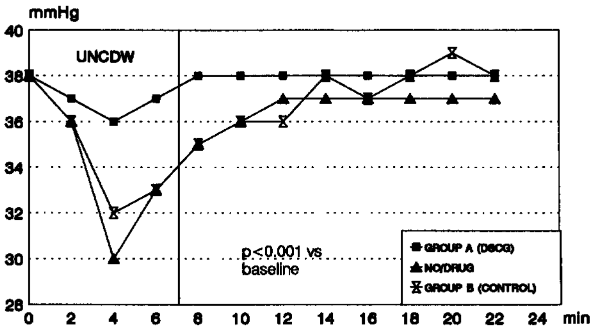

FIG. 1. Transcutaneous $tcpCO_2$ time courses in asthmatic children in basal conditions (no/drug, $n = 18$), in Group A children (DSCG, $n = 9$) and in Group B children (control, $n = 9$), during and after UNCDW inhalation (mean % values).

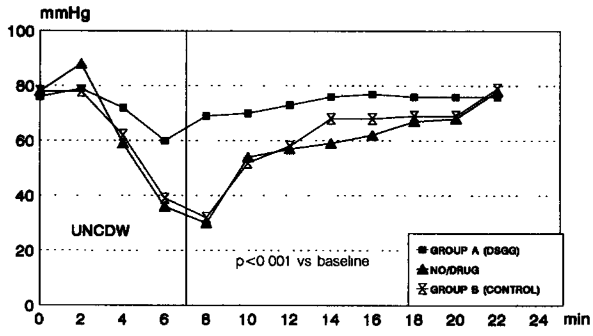

FIG. 2. Transcutaneous $tcpO_2$ time courses in asthmatic children in basal conditions (no/drug, $n = 18$), in Group A children (DSCG, $n = 9$) and in Group B children (control, $n = 9$), during and after UNCDW inhalation (mean % values).

tion in real time of their trends of variation, allowed us to identify the different bronchial structures involved in the hyper-reactive response.[8]

Furthermore, only in asthmatics does the bronchial challenge induce an appreciable degree of hypoxia with a later onset, shifted maximum effect, and longer duration, indicative of a mismatched ventilation/perfusion ratio.

We could verify that, unlike normal adult subjects,[8] asthmatics (also free of symptoms), had unusual time courses for $tcpO_2$ and $tcpCO_2$ during UNCDW, namely: (1) the first phase in which $tcpCO_2$ decreased immediately after the start of UNCDW exposure; and (2) the second phase in which $tcpO_2$ decreased at about the fourth min of inhalation, reaching the lowest value four min after the end of exposure.

It is reasonable to suggest that the initial drop of $tcpCO_2$ is linked to the response of the bronchial mucosa, the first target of the challenge. This allows us to evaluate the behaviour of the bronchial epithelium which is the seat of inflammation (hyper-reactivity).

The phase following this is the contraction of the smooth muscle, leading to bronchospasm, and then to the drop in $tcpO_2$ (hyper-responsivity).

A further demonstration of this behaviour is that the previous administration of a beta-2 agonist drug 10 min before the challenge was shown to prevent the occurrence of a hypoxic response to the fog (effect on the bronchial hyper-responsivity), while the hypocapnic response remained unchanged.[9,10]

The treatment with anti-inflammatory drugs showed (in adults) the gradual normalization of the time courses of $pCO_2$ (effect on bronchial hyper-reactivity).[11]

In this study, we obtained the same time courses of blood gases during the UNCDW test in children as had been observed in adults; on the other hand, after treatment with cromoglycate (Group A), a normalization of the time course for $pCO_2$ occurred and only a small decrease of $tcpO_2$ (not above 10% from baseline) was observed.

In conclusion, it emerges that: UNCDW induces nonspecific bronchoconstriction in asthmatic children, with a similar time course of blood gases to that of adults; the treatment with cromoglycate normalizes the time course of $pCO_2$ (hyper-reactivity) and reduces dramatically the drop in the $pO_2$ time course (hyper-responsivity) during and after the UNCDW test.

### References

1. Bernstein IZ. Cromolyn sodium in the treatment of asthma: coming of age in the United States. *J Allergy Clin Immunol* 1985; **76:** 381.
2. Griffin MP, MacDonald N, McFadden ER. Short- and long-term effects of cromolyn sodium on the airway reactivity of asthmatics. *J Allergy Clin Immunol* 1983; **71:** 331.
3. Allegra L, Bianco S. Non-specific bronchoreactivity obtained with an ultrasonic aerosol of distilled water. *Eur J Respir Dis* 1980; **61** (Suppl 106): 41–49.
4. Mappa L, Muggeo PG, Torio D, Salerno R, Rigillo N. Bronchial challenge by ultrasonically nebulized 'cold' distilled water (UNCDW) in children with asthma. *Eur Respir J* Sept 1993; **6** (Suppl 17): 615s–P1980.
5. Dal Negro RW, Zoccatelli O, Turco P, Pomari C, Serra R. Transcutaneous $O_2$ and $CO_2$ monitoring in the evaluation of non-specific bronchial hyper-reactivity. *SEP Newsletter No. 11.* Banbury, Oxford: Cheney and Sons, 1987; 5–9.
6. Anderson SD, Schoeffel RE, Finney M. Evaluation of ultrasonically nebulized solution for provocation testing in patients with asthma. *Thorax* 1983; **38:** 284–291.
7. Armitage P. Statistica medica. In: *Metodi Statistici per la Ricerca in Medicina* (5th ed.) Milan: Feltrinelli Ed: 1982; 188–212.
8. Dal Negro R, Allegra L. Blood gas changes during and after nonspecific airway challenge in asthmatic and normal subject. *J Appl Physiol* 1989; 2627–2630.
9. Dal Negro R. Abnormal airways responses in atopy. *Triangle* 1988; **27:** 3.
10. Turco P, Dal Negro R, Cogo A, Allegra L. Changes in $tcCO_2$ time course due to ultrasonically nebulized distilled water (UNDW) in atopics before and after salbutamol. *Eur Respir J* 1989; **2** (Suppl): 8 (abstract).
11. Dal Negro R. La transizione tra normalità e risposta iperrattiva. *Ital J Chest Dis* 1989; **43** (Suppl 3): 213–218.
